# Assessment of Foot Health and Toe Strength in Older Adults Undergoing Heart Valve Surgery: A Pilot Study

**DOI:** 10.3390/healthcare14081090

**Published:** 2026-04-20

**Authors:** Hiromi Moriwaki, Mihoko Ishizawa

**Affiliations:** Faculty of Nursing, Nara Medical University, Nara 634-0813, Japan

**Keywords:** heart valve surgery, foot and nail conditions, foot strength

## Abstract

**Objectives**: We aimed to explore foot condition and toe strength in older adults undergoing heart valve surgery. **Materials and Methods**: This exploratory pilot study included nine older adults undergoing heart valve surgery. Subjective data on foot-related symptoms, self-care status, nail care, footwear, exercise habits, and fall history were collected. Preoperative foot and nail conditions were assessed using observation and photography. Toe strength was measured preoperatively in all participants and postoperatively in a subset of participants when feasible. Descriptive analyses were primarily conducted, with exploratory group comparisons. **Results**: Participants frequently reported foot-related symptoms and difficulties with foot self-care prior to hospitalization. Lower toe strength appeared to be related to greater difficulties in foot self-care, whereas higher toe strength was more commonly observed in those reporting regular exercise habits. Postoperative toe strength was reassessed in six participants. In a participant with prolonged intensive care unit (ICU) stay, delayed recovery of toe strength was observed. **Conclusions**: These preliminary findings suggest that foot condition and toe strength may be relevant to physical function and fall prevention-related factors in older a dults undergoing cardiac surgery. Reduced toe strength may be related to self-care difficulties, and prolonged ICU stay may influence the recovery of toe strength. However, due to the small sample size, these findings should be interpreted as exploratory and hypothesis-generating.

## 1. Introduction

Many older adults have foot abnormalities [1]. A previous study examining 102 older individuals reported that 80.4% required medical intervention [2]. Age-related physiological changes cause the feet to assume a rotated posture due to increased stiffness of soft tissues, muscle weakness, and reduced range of motion [3]. These changes increase the risk of structural deformities such as hallux valgus.

Falls are a major concern in older adults, and previous studies have reported a high incidence of falls in this population [4,5]. Older individuals with a history of multiple falls have been shown to have a higher prevalence of foot abnormalities compared with those without such a history [6]. In addition, foot abnormalities are associated with impaired postural balance, which further increases the risk of falls. These findings suggest that foot problems, including thickened toenails and hallux valgus, may contribute to decreased walking ability and increased fall risk [7,8].

With the rapid aging of the global population [8,9], fall prevention in hospitalized older adults has become an increasingly important issue. Previous research has shown that foot care can improve postural control in older individuals [10], highlighting the importance of assessing and managing foot health as part of fall prevention strategies. However, podiatric interventions for fall prevention have not yet been fully recognized or widely implemented [11,12].

Patients undergoing cardiovascular surgery are at particularly high risk of falls. Previous studies have identified factors such as the use of vasopressors and analgesics, arrhythmias, blood pressure fluctuations, and delirium as contributing to this risk [13,14,15]. However, no studies have specifically focused on foot conditions as an independent risk factor for falls in this population.

Furthermore, in Japan, podiatric education programs are limited, and routine foot care is not widely implemented. While foot care for patients with diabetes has been covered by medical insurance since 2008, awareness of foot care for other conditions remains low. As a result, the role of foot health in hospitalized patients has not been sufficiently explored.

Therefore, this study aimed to evaluate foot health and toe strength in patients undergoing cardiovascular surgery. The findings may provide a basis for developing effective foot care strategies to prevent falls in this population.

## 2. Materials and Methods

### 2.1. Patients and Selection Criteria

This study included patients aged ≥65 years who were admitted to Hospital A for scheduled heart valve surgery. The target population was limited to patients undergoing cardiac surgery that did not involve surgical wounds on the lower extremities. Patients who were unable to undergo foot assessment or provide informed consent were excluded.

### 2.2. Measurement Period

Data were collected between August 2023 and August 2024.

### 2.3. Data Collection

Data were obtained from two distinct sources: electronic medical records and structured interviews. Toe strength was measured preoperatively in all participants. Postoperative toe strength was reassessed in a subset of participants (*n* = 6) during the early recovery period, when feasible depending on clinical constraints such as postoperative condition, early discharge, or patient fatigue.

#### 2.3.1. Electronic Medical Records

Clinical information, including age, sex, diagnosis, surgical procedure, comorbidities, and length of ICU stay, was obtained from electronic medical records.

#### 2.3.2. Interview Details

Information on subjective foot-related symptoms, foot self-care practices, nail care status, usual footwear, exercise habits, and history of falls was collected through structured interviews.

#### 2.3.3. Observation and Measurement Details

Toe strength was assessed preoperatively.

Foot and nail conditions: The foot (skin and shape) and nail conditions were assessed via gross observation and photography using predefined criteria. All assessments were conducted by a single trained assessor who received prior instruction in the evaluation procedures. The presence of edema was determined if an indentation remained after applying pressure on the dorsal surface of the foot and the anterior tibia using the thumb for 10 s.

For hallux valgus, an angle of 20–30° was defined as mild, 30–40° as moderate, and ≥40° as severe. Digitus minimus varus was defined as an angle of ≥10° between the fifth metatarsal and the proximal phalanx; severity was classified as mild (<20°), moderate (20–30°), and severe (>30°). Hammertoe was defined as flexion deformity at the proximal interphalangeal (PIP) joint of the second to fifth toes. These deformities were assessed based on visual inspection and photographic evaluation according to the predefined criteria by the same assessor.

**Figure 1:**
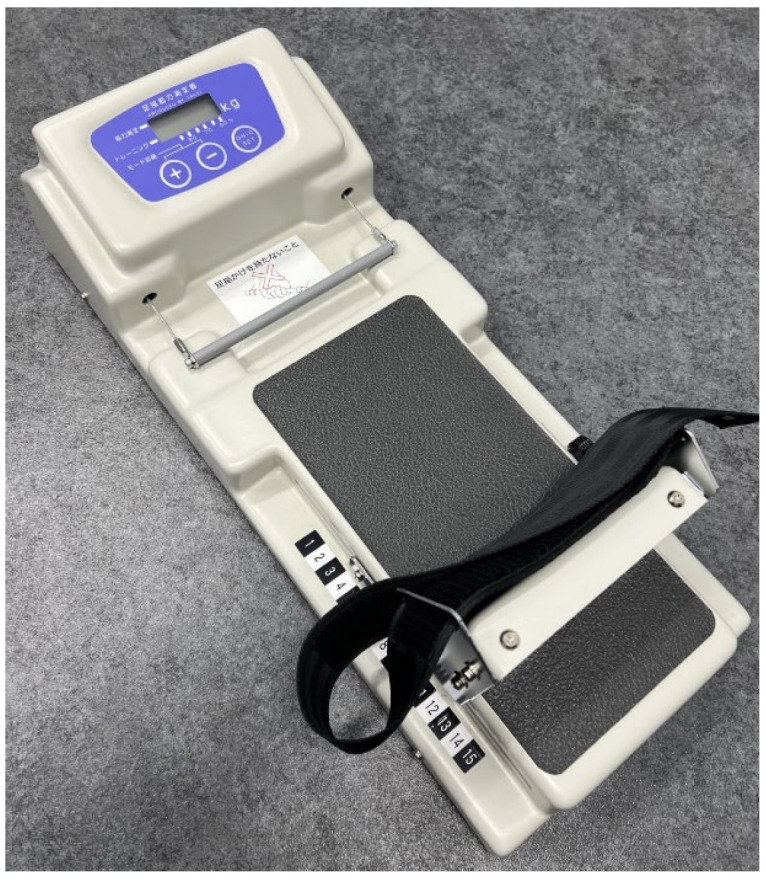
Toe-muscle strength-measuring device (Takei Corporation, Tokyo, Japan).

Foot function: Toe strength was assessed using a toe-muscle strength-measuring device (Takei Corporation, Tokyo, Japan) (Figure 1), which quantifies the force exerted by the toe muscles. This device has been used in previous rehabilitation research [16,17] and is considered safe and easy to use.

During measurement, participants were seated on a bed adjusted so that the knee and ankle joints were positioned at approximately 90°. The toes were placed on a bar, the foot position was stabilized, and participants were instructed to pull the bar using their toes. Two measurements were taken on each side, and the higher value was used for analysis.

### 2.4. Analytical Methods

The case characteristics obtained from the survey were summarized descriptively using counts and percentages. Participants were categorized into standard and substandard toe strength groups based on reference values from previous study [18] (6.5–18.3 kg for men and 4.2–7.8 kg for women over 50 years of age).

Due to the small sample size and the exploratory nature of this pilot study, no inferential statistical tests were performed. Observed patterns and trends regarding toenail condition, exercise habits, and self-care difficulties were described narratively rather than statistically analyzed.

### 2.5. Ethical Considerations

This study was conducted in accordance with the principles of the Declaration of Helsinki and the recommendations of the International Committee of Medical Journal Editors (ICMJE). Ethical approval was obtained from the Medical Ethics Review Committee of Nara Medical University (Approval No. 3569; date of approval: [4 July 2023]). All procedures complied with relevant laws and institutional guidelines.

Participants were fully informed, both in writing and verbally, about the nature and purpose of the study, their right to decline participation or withdraw at any time, and the measures taken to ensure confidentiality. Written informed consent was obtained from all participants prior to inclusion in the study. To protect participant privacy, all data were anonymized and coded to prevent identification, used solely for research purposes, and securely stored according to institutional data protection policies.

Given the small sample size, additional care was taken to ensure that no individual could be identified from the reported results.

## 3. Results

### 3.1. Basic Demographics of the Patients (Table 1)

Nine patients (six men and three women) were included in this study. The age distribution included one patient in their 60 s, four in their 70 s, and four in their 80 s. Eligibility was limited to patients aged ≥65 years undergoing heart valve surgery, which contributed to the small sample size.

Primary diagnoses and surgical procedures are summarized in Table 1. Aortic stenosis, aortic regurgitation, mitral regurgitation, and thoracic aortic aneurysm were observed as underlying conditions, and surgical procedures included aortic valve replacement, coronary artery bypass grafting, graft replacement, mitral annuloplasty, and arrhythmia surgery.

**Table 1:** Participant characteristics.

	Age	Sex	Diagnosis	Surgical Procedures
A	76	F	AS	AVR; CABG
B	71	M	AS	AVR
C	83	F	TAA; AS	GR; AVR
D	81	F	MR	MAP
E	74	M	AS	AVR; Arrhythmia surgery
F	83	M	AR	AVR
G	65	M	AS	GR; AVR
H	80	M	AR	AVR; Arrhythmia surgery
I	77	M	MR; AS	AVR; MAP

Abbreviations: AS, aortic stenosis; AR, aortic regurgitation; MR, mitral regurgitation; TAA, thoracic aortic aneurysm; AVR, aortic valve replacement; CABG, coronary artery bypass grafting; GR, graft replacement; MAP, mitral annuloplasty.

### 3.2. Subjective Foot Symptoms, Self-Care Status, Exercise Habits, and History of Falls (Table 2)

Participants exhibited varying degrees of foot-related symptoms and self-care abilities. Several participants reported difficulties in maintaining foot hygiene, often in the context of underlying health conditions such as heart failure, arrhythmia, or recurrent loss of consciousness. These difficulties were reflected in reduced bathing frequency, low awareness of foot care, or inability to trim nails. For example, some participants avoided cutting long nails due to fear or physical limitations.

**Table 2:** Subjective foot symptoms, self-care status, and history of falls.

	Foot/Nail Symptoms & Self-Care	Nail Care	Exercise Habits
A	Edema, dirty legs, difficulty washing	Delayed due to swelling	Minimal, goes out
B	None, washes feet carefully	Cut every—3 weeks	Daily walking
C	Difficult foot washing for 1 month, moisturizing	Regular	Minimal movement
D	Long nails, callus	Avoids cutting	Minimal, ankle exercises
E	Hard to wash due to illness & fainting	Cut when grows	Minimal since arrhythmia
F	Feet washed normally	Cut when grows	No specific habits
G	Dry in winter, onychomycosis	Cut when grows	Tennis, golf, walking
H	Athlete’s foot, misshapen, callus	Cut when grows	Mostly at home
I	Difficult foot washing due to dialysis/stiffness	Managed to cut	Mostly at home

### 3.3. Preoperative Foot Shape, Foot Condition, and Nail Status (Table 3, Figure 2)

#### 3.3.1. Foot Shape

All participants exhibited mild-to-moderate hallux valgus, suggesting that forefoot deformity was common in this sample. In addition, one participant presented with hammertoes and another with severe hallux valgus, indicating that more pronounced deformities were present in a limited number of cases.

**Figure 2:**
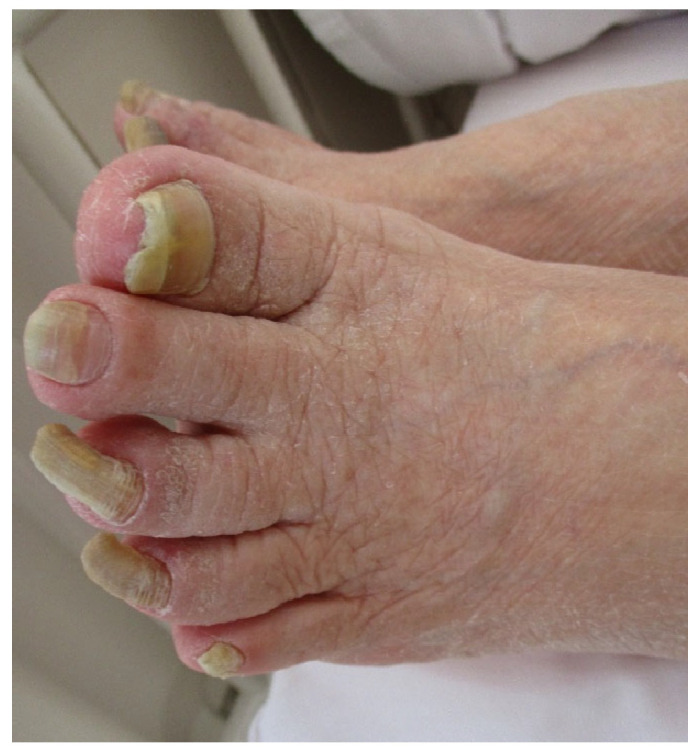
Subject‘s foot.

**Table 3:** Preoperative condition of the foot and foot strength.

	Foot/Nail Status	Toe Strength (R/L, kg)
A	HV(L), DMV(M), HT; Dry, Callus, Swell; Onycho	2.1/3.4
B	HV(L), DMV(M); Red 5th toe; Normal nails	11.8/8.0
C	DMV(M); Red, tinea-like 4th toe; Normal nails	2.5/2.5
D	HV(L), DMV(L); Dry, heel keratin, Swell; Onycho, Onychogryphosis, Ingrown nail	2.8/3.3
E	HV(M), DMV(L); Dry, heel keratin, Callus 1st toe; Leuconychia	6.1/6.4
F	DMV(M); Dry, Swell, Cold sens; Onychogryphosis	1.7/1.4
G	HV(M), DMV(M); Plantar calluses; Leuconychia	16.7/18.7
H	HV(M), DMV(M); Plantar calluses; Leuconychia	7.1/6.5
I	HV(M), DMV(M); Dry; Hyperkeratosis	8.9/8.8

Legend: HV = Hallux valgus; DMV = Digitus minimus varus; HT = Hammertoe; Dry = Dryness of skin; Callus = Callus formation; Swell = Edema or swelling; Onycho = Onychomycosis; Leuconychia = White discoloration of nails; Cold sens = Sensitivity to cold.

#### 3.3.2. Foot Skin Condition

Dry skin was observed in over half of the participants, while swelling and cold sensation were less frequent. Localized calluses, erythema, and keratinization were observed in several participants, particularly in weight-bearing areas such as the heels and toes. These findings indicate that impaired skin condition, including dryness and hyperkeratosis, was commonly observed preoperatively in this sample.

#### 3.3.3. Nail Condition

Nail abnormalities varied across participants, including normal nails, tinea-like changes, onychogryphosis, ingrown nails, and linear nail discoloration (leuconychia). Several participants exhibited fungal-like nail changes or hyperkeratosis, which may reflect inadequate foot care and/or chronic mechanical stress. These observations highlight the importance of careful preoperative assessment.

Toe strength measurements showed considerable variability among participants. Some individuals demonstrated reduced strength in one or both feet, whereas others maintained relatively higher strength. These findings may suggest that structural and skin or nail conditions could be accompanied by functional differences; however, this should be interpreted cautiously given the small sample size.

### 3.4. Toe Strength and Its Association with Foot Condition and Self-Care (Table 4, Figure 3)

Toe strength measurements were obtained from six participants (cases A, B, D, E, G, and I), as three participants (C, F, and H) could not complete assessments due to clinical constraints, including postoperative condition. In all measured cases, toe strength returned to preoperative levels during the recovery period, although the duration varied. Case E required a longer recovery period (11–16 days).

Exploratory comparisons between participants with relatively higher and lower toe strength suggested potential differences in foot-related characteristics. In this small sample, participants with lower toe strength appeared more likely to exhibit dry skin, swelling, cold sensitivity, tinea-like nails, calluses, nail abnormalities, hallux valgus, lack of exercise habits, and difficulties in self-care. In contrast, those with relatively higher toe strength tended to show fewer such features and were more likely to report regular exercise habits.

These observations may suggest a possible relationship between toe strength and aspects of foot health, physical activity, and self-care. However, given the very small sample size (*n* = 9; *n* = 6 for some analyses), these findings should be interpreted as exploratory and hypothesis-generating rather than conclusive.

**Figure 3:**
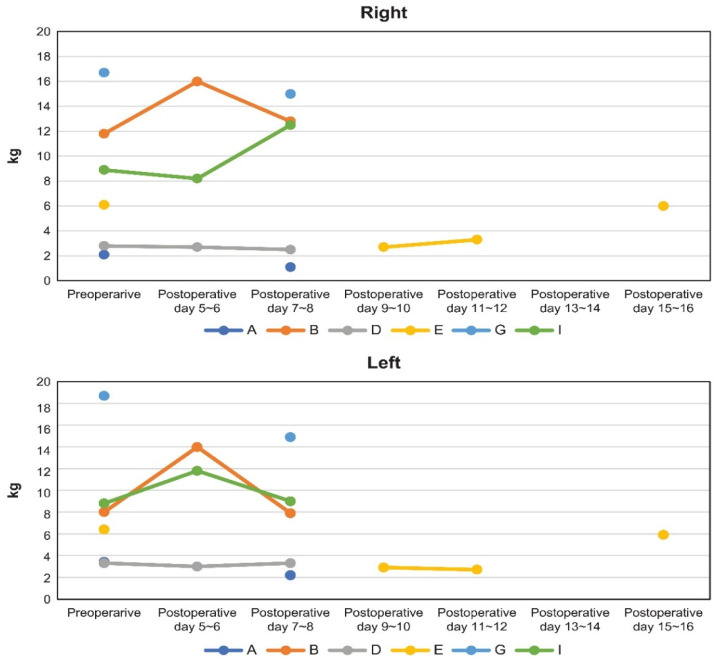
Postoperative toe strength.

**Table 4:** Comparison of toenail condition, self-care status, and toe strength between the groups.

	Toe Strength Standard Value Group (*n* = 4)	Toe Strength Substandard Value Group (*n* = 5)
Dryness (Yes/No)	1/3	4/1
Sensitivity to cold (Yes/No)	1/3	2/3
Swelling (Yes/No)	1/3	3/2
Tinea-like (Yes/No)	0/4	2/3
Calluses (Yes/No)	1/3	2/3
Nail abnormalities (Yes/No)	3/1	4/1
Hallux valgus (Yes/No)	4/0	3/2
Exercise habit (Yes/No)	2/2	0/5
Self-care difficulties(Yes/No)	0/4	4/1

## 4. Discussion

### 4.1. Toenail Status of Older Adults Undergoing Heart Valve Surgery

The present study indicates that older adults undergoing heart valve surgery frequently exhibit foot and toenail problems at hospital admission. Many participants had limited self-care ability, with difficulties in washing their feet or trimming nails, despite some awareness of foot hygiene. This limited engagement in foot care may be associated with the presence of foot conditions such as dryness, edema, calluses, and nail abnormalities observed in this sample (Table 2).

Previous studies have reported a high prevalence of foot disorders in older adults, including onychomycosis, and have described age-related foot deformities and a generally high burden of foot and ankle conditions [19,20,21], which is consistent with the present findings. In addition, inadequate footwear and limited physical activity may exacerbate these conditions and may be associated with reduced mobility and increased fall risk.

In Japan, podiatric care is not yet widely established, and routine foot assessment is not systematically integrated into standard care. This may partially contribute to the persistence of untreated conditions such as calluses, corns, and ingrown nails. Future research should explore strategies to improve awareness of foot care, including appropriate footwear selection, exercise promotion, and systematic foot assessment in older adults undergoing cardiac surgery.

### 4.2. Preoperative Toe Strength and Toenail Condition, Self-Care Difficulties, and Exercise Habits

Participants with regular exercise habits in this study tended to demonstrate higher toe strength compared with those without such habits, which is consistent with previous reports suggesting that toe exercises may improve toe muscle function [22,23].

No clear relationship was observed between specific toenail conditions and toe strength in this small sample. However, participants who reported difficulties in foot self-care appeared to more often exhibit lower toe strength. For example, in one case, avoidance of toenail trimming due to fear and limited mobility was accompanied by nail thickening (onychogryphosis). These observations may suggest that reduced physical function and limited mobility could be related to both self-care ability and toe strength; however, these findings should be interpreted cautiously given the small sample size.

Disease-related symptoms prior to surgery, such as fatigue, edema, or arrhythmia (cases A, C, and E), may also have influenced daily activities, including foot care and physical activity. Among the participants, approximately half demonstrated toe strength below age-adjusted reference values, suggesting possible functional decline in this population.

Reduced toe strength may have functional implications. Previous studies have reported that toe and forefoot muscle weakness may be associated with impaired walking speed, balance, and postural stability [5,24,25], and that hallux and lesser toe weakness may be related to reduced mobility [26,27]. Therefore, older adults undergoing heart valve surgery may be at increased risk of functional decline; however, the present findings should be interpreted as exploratory due to the small sample size (*n* = 9; *n* = 6 for some analyses).

Clinically, the assessment of foot condition and toe strength at admission may be useful as part of comprehensive geriatric evaluation. Early attention to foot care and referral to appropriate specialists may support postoperative functional management.

### 4.3. Postoperative Changes in Toe Strength and Recovery Patterns

In this study, one participant with prolonged ICU stay showed delayed recovery of toe strength compared with other participants. This may reflect the effects of prolonged immobilization, reduced physical activity, and general deconditioning during intensive care.

Among the six participants assessed postoperatively, surgical duration ranged from 5 to 10 h, and ICU stay ranged from 3 to 7 days. Heart valve surgery in older adults typically requires relatively long operative and postoperative recovery periods, which may influence early functional recovery.

All participants eventually regained preoperative toe strength during the postoperative period with rehabilitation support. However, in one case (case E), recovery to preoperative levels required 16 days, and this participant also had the longest ICU stay (7 days). Although the average ICU stay for cardiac surgery patients in Japan is reported to be approximately 2–3 days [28], prolonged ICU stay in this case may have been associated with delayed recovery; however, this observation is limited to a single case.

Post-intensive care syndrome (PICS), characterized by physical, cognitive, and psychological impairments after ICU care, has been reported in a substantial proportion of ICU survivors, and longer ICU stays may increase this risk [29]. ICU-related factors may therefore be relevant when considering postoperative functional outcomes, including mobility and fall risk.

In addition, frailty, comorbidities, and overall postoperative recovery status may influence toe strength recovery. These factors were not controlled for in the present study and should be examined in future research.

Toe strength has been suggested to be related to balance and gait stability. Footwear has also been reported to be associated with postural stability and fall risk in older adults [30,31]. Therefore, a multifactorial approach considering physical function, self-care ability, and appropriate footwear may be important in fall prevention strategies.

However, given the exploratory nature of this study and the small sample size, these findings should be interpreted with caution. Further studies with larger samples are needed to clarify these relationships.

## 5. Conclusions

This exploratory pilot study suggests that older adults undergoing heart valve surgery may present with foot problems and reduced toe strength prior to surgery. Toe strength may be related to self-care ability and exercise habits, and the postoperative recovery of toe strength may be influenced by clinical factors such as ICU stay.

However, due to the small sample size and exploratory design, these findings should be interpreted as preliminary and hypothesis-generating. Further studies with larger sample sizes and more rigorous methodology are needed to clarify these relationships and to explore potential interventions targeting foot health and physical function in this population.

## 6. Study Limitations

This study has several limitations. First, the small sample size and single-center design limit the generalizability of the findings. In addition, the study population was restricted to older adults undergoing heart valve surgery, which may introduce selection bias.

Second, postoperative toe strength measurements could not be obtained in all participants due to unstable clinical conditions, potentially leading to missing data bias.

Third, the timing of postoperative measurements could not be standardized because of differences in patient recovery status, resulting in variability in assessment timing. Therefore, it was not possible to evaluate changes in toe strength based on a uniform postoperative timeline.

Finally, the cross-sectional nature of some assessments, the small sample size, and the reliance on self-reported data (e.g., self-care status and symptoms) limit the ability to infer causal relationships or perform inferential statistical analyses.

## Data Availability

The datasets used and/or analysed during the current study are available from the corresponding author on reasonable request.
